# Multiscale modelling of hydraulic conductivity in vuggy porous media

**DOI:** 10.1098/rspa.2013.0383

**Published:** 2014-02-08

**Authors:** K. R. Daly, T. Roose

**Affiliations:** School of Engineering Science, University of Southampton, Southampton SO17 1BJ, UK

**Keywords:** multiscale modelling, image-based modelling, X-ray Computer Tomography, soil water flow

## Abstract

Flow in both saturated and non-saturated vuggy porous media, i.e. soil, is inherently multiscale. The complex microporous structure of the soil aggregates and the wider vugs provides a multitude of flow pathways and has received significant attention from the X-ray computed tomography (CT) community with a constant drive to image at higher resolution. Using multiscale homogenization, we derive averaged equations to study the effects of the microscale structure on the macroscopic flow. The averaged model captures the underlying geometry through a series of cell problems and is verified through direct comparison to numerical simulations of the full structure. These methods offer significant reductions in computation time and allow us to perform three-dimensional calculations with complex geometries on a desktop PC. The results show that the surface roughness of the aggregate has a significantly greater effect on the flow than the microstructure within the aggregate. Hence, this is the region in which the resolution of X-ray CT for image-based modelling has the greatest impact.

## 1. Introduction

The macroscopic Darcy's law and Richard's equation which describe flow in porous media can be derived either using formal two-stage homogenization [1–4], two-scale convergence [4,5] or volume-averaging techniques [6,7]. Homogenization has been widely used to describe flow in single porosity materials [1,4,8,9] and, more recently, to describe flow and diffusion in dual porosity models where the substructure is composed of two different domains with different porosity [10,11]. In these structures, Darcy's law is applied in each domain and homogenization allows an averaged Darcy law for single-phase flow or Richard's equation for two-phase flow to be derived [10,12,13].

Another related structure is vuggy porous media [14–17]. Vuggy porous materials consist of regions of tightly packed microparticles which form aggregates separated by larger pores or vugs. In the vuggular region, Darcy's law is no longer applicable and Stokes flow must be considered [15]. The macroscopic behaviour of flow in such a medium relates not only to the flow in the vugs and the aggregate, but also to the condition applied at their interface. This condition is geometry-dependent and is often described using the Beavers and Joseph condition [15], or the Saffman approximation to the Beavers and Joseph condition [18]. Both these conditions are slip boundary conditions which relate the shear stress to the velocity at the surface of a porous medium with slip length proportional to the square route of the hydraulic conductivity in the porous medium, where the constant of proportionality is left as an experimentally determined fitting parameter.

There have been several studies which aim to eliminate the fitting parameters used in the Beavers and Joseph conditions [14,19–22]. Levy & Sanchez-Palencia [14] derived simplified boundary conditions for two limiting cases: the case where pressure gradients were normal and non-normal to the interface. The rigorous derivation of the Beavers and Joseph condition, which generalizes these cases, was first derived by Jäger & Mikelic [19]. This method uses the assumption that the porous domain is periodic in the direction tangential to the boundary enabling the Beavers and Joseph coefficient to be derived through a cell problem relating the average velocity in the Stokes domain to a unit shear stress applied at the boundary.

Recent studies by Arbogast *et al.* consider vuggy porous geometries [16,17]. Arbogast *et al.* apply the Saffman approximation of the Beavers and Joseph condition on the boundary between the vug and the adjacent porous medium [15,18]. This condition is based on the fitting parameter in the Beavers and Joseph condition which depends on the exact geometry of the boundary considered. The result of these studies is a macroscopic derivation of Darcy's law in which the hydraulic conductivity depends on the coupled flow in the vugs and the aggregate.

In this paper, we extend the work of Arbogast to include the geometrical properties of the interface between the vugular region and the porous region. Specifically, we study the flow of fluid in vuggy porous media in the context of two-phase flow in soils. In order to answer fundamental questions regarding flow in porous media and the interaction of these flows with external sources and sinks, e.g. roots, it is essential to develop a model which captures all necessary geometrical features of the soil [23]. Not only will this model provide significant insight into the flow mechanisms and advanced models which can be incorporated into image-based simulations [24], it will also feedback into the resolution driven imaging of soils through X-ray computed tomography (CT) and synchrotron radiation-based microtomography [25,26] by providing a lower limit to the scale of soil features which affect flow properties and hence, need to be detected by X-ray CT.

**Figure 1.**
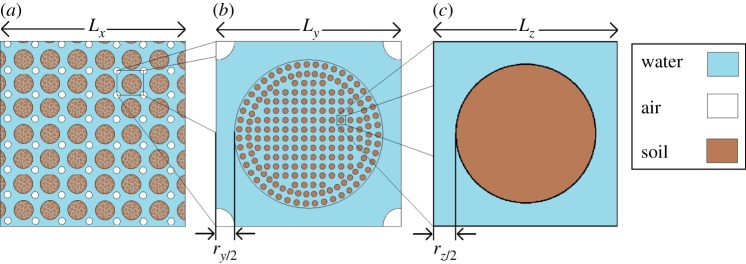
Idealized soil schematic showing typical length scales and pore sizes. (*a*) Shows the macroscopic picture, a periodic arrangement of aggregates and air bubbles, (*b*) shows a zoomed in image of the aggregate scale, this is a single unit cell of the macroscale geometry showing the internal structure of the aggregate and (*c*) shows a zoomed in image of the microscale geometry inside the aggregate. (Online version in colour.)

We consider the flow of air and water in a periodic array of soil aggregates (figure 1*a*). The aggregates are composed of a periodic array of soil particles (figure 1*b*). The resulting geometry has three different scales: the soil particle scale, the aggregate scale and the macroscopic or field scale. This structure has been designed to have a bimodal pore size distribution as observed in measurements on typical soils [27–29]. The hydraulic properties of this geometry are highly complex. To simplify the hydraulic properties of the fluids, we consider the case where the aggregate is completely hydrophilic and the interface between the two fluids is stationary [4]. The dynamics of fluids in vuggy porous media are highly complex [30–33]. These geometries exhibit circulation near interface between the vug and the porous region even in the case of low Reynolds numbers [33], this is attributed to flow penetration from the vug into the porous region [34,35]. However, these effects are seen to decay over time and settle to the steady-state flow properties [33]. We consider the commonly used steady-state Stokes equations, [4,16,17], and neglect the short time scale dynamics of the fluid. To understand the precise role of geometry in such a structure, we apply the boundary conditions of Jäger & Mikelic [19–22]. Our resulting model is free from fitting parameters and describes the flow in vuggy porous media defined entirely by the geometry and fluid properties.

The application of this theory requires the assumption of periodicity of the pore structure on the aggregate surface [19], something which is not easy to achieve even in idealized geometries. However, the results for hydraulic conductivity are shown to be accurate in the cases tested. Typical errors for the geometry shown in figure 1 are 2% for the Beavers and Joseph condition and 10% for the Saffman condition for a highly porous aggregate with pore sizes ≈23% the size of the maximum space between aggregates. The errors reduce to 1% for the Beavers and Joseph condition and 2% for the Saffman condition in the case of a low porosity aggregate with typical pore size of ≈8% the size of the space between the aggregates. In non-ideal soil geometries, the assumption of periodicity is less likely to be accurate. However, this can be addressed by considering successively larger sample sizes and imposing periodicity. As the sample size increases the errors induced by imposing periodicity are likely to reduce and the macroscopic properties will converge. The resulting hydraulic conductivity depends on the underlying periodic structure of the aggregate, the nature of the flow at the aggregate surface and the flow around the aggregate itself. It is shown that the required boundary conditions depend on the relative scales of the inter-aggregate pores, the vug radius and the macroscopic sample size.

This paper is arranged as follows: in §2, the theory is derived and for flow in vuggy porous media. In §3, the boundary conditions tested for two- and three-dimensional idealized aggregates. Finally, the results are discussed with reference to imaging in §4.

## 2. Model

The derivation of the model is arranged as follows: first, in §2*a*, we derive the scaled equations governing fluid flow in the porous medium. In §2*b*, we apply homogenization theory to the equations and study the behaviour of flow in an individual soil aggregate. In §2*c*, we study the boundary conditions at the aggregate surface in terms of the theory developed by Jäger & Mikelic [19,20]. Finally, in §2*d*, we derive the macroscopic equations which describe the behaviour of the averaged flow in the soil while taking the microstructure into account.

### (a) Scaling

We consider the flow of two incompressible fluids contained within the porous medium (figure 1) with length scales and fluid properties applicable to the flow of water and air in soil [36,37]. The soil consists of a periodic collection of aggregates (figure 1*a*) with an internal microstructure which is also periodic (figure 1*b*). We refer to the flow around a collection of aggregates as the aggregate scale and the flow through the pore structure within the aggregate as the microscale. The structure is porous on both the aggregate scale and the microscale with typical pore size *r*_*y*_ and *r*_*z*_, respectively. The aggregate itself (figure 1*b*) is assumed to be roughly periodic both with distance around the surface and internally. This assumption is clearly not completely valid even for simple geometries such as this one and for simulations carried out on X-ray CT geometries it is necessary to increase the size of the periodic unit considered until the hydraulic properties converge. The structure has fundamental period *L*_*y*_ on the aggregate scale and *L*_*z*_ on the microscale. The macroscopic length scale, *L*_*x*_ (figure 1*a*) is chosen based on the macroscopic geometry considered. Here, we consider the case of *L*_*x*_≈10 cm, a length comparable with the typical length scale for soil columns used in X-ray CT imaging [26]. The maximum inter-aggregate pore size is typically *r*_*y*_≈100 μm [29]. In unsaturated soils with no air sources or sinks, the dominant force is gravity. For gravity-driven Poiseulle flow, we find that the Reynolds numbers for the water and air phases are
2.1
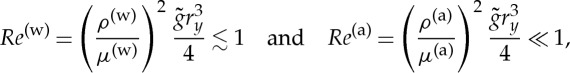
where *μ*^(w)^ and *μ*\^(a)^ are the water and air viscosity, *ρ*^(w)^ and *ρ*^(a)^ the water and air density and 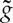 is the acceleration because of gravity. Here, we concentrate on highly tortuous porous media and as such we expect this to be an over-estimate of the Reynolds number. Hence, we consider the Stokes limit of the Navier–Stokes equations.

We also assume that the system is in dynamic equilibrium such that the location of the boundary between the two different fluid phases is known/fixed. We denote the water domain *Ω*_w_, the air domain *Ω*_a_, the soil particle boundary *Γ*_s_ and the air–water interface *Γ*_aw_. The dimensional Stokes equations, in an unsaturated geometry [4], are
2.2a
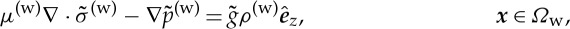

2.2b
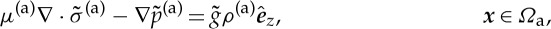

2.2c
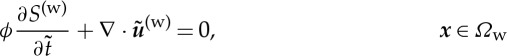

and
2.2d
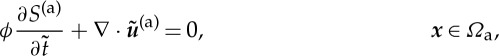
where 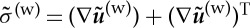 and 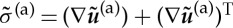 are the stress tensors for the fluid phases, 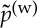 and 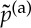 are the water and air pressures, respectively, 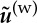 and 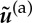 are the water and air velocities, *S*^(w)^ and *S*^(a)^ are the water and air saturation which satisfy *S*^(w)^+*S*^(a)^=1 and *ϕ* is the porosity denoting the total fraction of volume available for flow. We note that equations (2.2*c*,*d*) are macroscopic equations which refer to the saturation of the soil. The rigorous derivation of these equations requires careful consideration of the fluid–fluid interface dynamics and is beyond the scope of this paper. The macroscopic equations we have used are commonly accepted in the homogenization of two fluids in porous media [4] and, as we will show in §2*b*, on the microscale reduce to the standard incompressibility conditions for each fluid. The porosity, *ϕ*, is also a macroscopic quantity and refers to the total fraction of space available for flow of either fluid. Assuming periodicity of the structure and that the soil matrix itself remains stationary then *ϕ* does not change over space or time. On the soil particle surface we use a no slip condition combined with zero fluid penetration. Hence, all the velocity components vanish on the surface:
2.2e
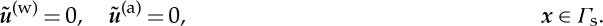
We also define a set of boundary conditions on the air–water interface. Specifically, we require that the interface is stationary, i.e. the normal velocity of the two phases is zero
2.2f
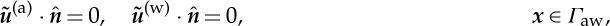
the slip length associated with tangential stress goes to zero for Stokes flow, hence, the tangential velocity is continuous
2.2g
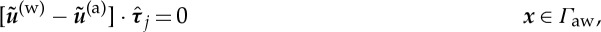
and there is a jump in the normal stress given by the surface tension curvature product
2.2h
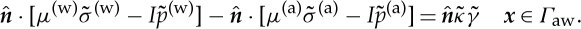
Here, 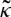 and 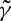 are the curvature and surface tension of the air–water interface, respectively; 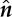 and 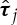, for *j*={1,2}, are the vectors normal and tangent to the air–water interface, respectively, with 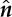 pointing into the water domain. We scale space to the fundamental period on the aggregate scale (figure 1*b*), 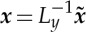 such that the aggregate scale is periodic with period 1. We define two small parameters, *ϵ*=*L*_*y*_/*L*_*x*_ denotes the ratio of the aggregate scale to the macroscopic length scale and *η*=*L*_*z*_/*L*_*y*_ denotes the ratio of the microscopic length scale to the aggregate length scale such that, locally to the aggregate, the microscale is periodic with period *η*. We introduce the following non-dimensional variables:

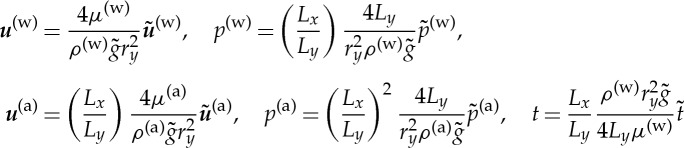
and the dimensionless parameters are

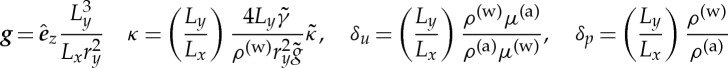
which we use to derive the non-dimensional equations prior to using multiple scale homogenization to derive the equations macroscopic which describe the flow of air and water in soil. This scaling results in equations which are effectively the same as the ones used in [4]. The key differences are that we have chosen to scale the viscosity and density of the two fluids into the parameters *δ*_*p*_ and *δ*_*u*_ and that we have chosen to scale with the aggregate length scale *L*_*y*_. This choice of spatial scaling results in a slightly different expansion of the gradient operator, we consider variations on the *L*_*x*_ scale small rather than considering variations on the *L*_*y*_ scale large. However, this choice seems a more natural as it makes it easier to keep track of the different spatial scales. The non-dimensional Stokes equations which results from this scaling are
2.3a
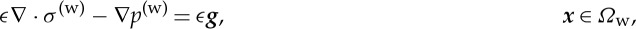

2.3b
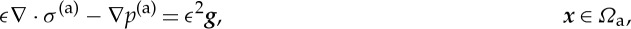

2.3c
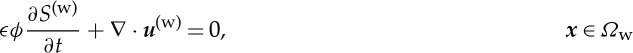

and
2.3d
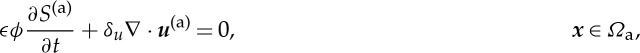
with boundary conditions
2.3e
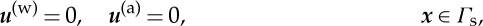

2.3f
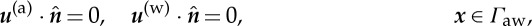

2.3g
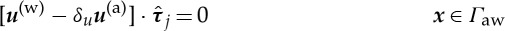

and
2.3h
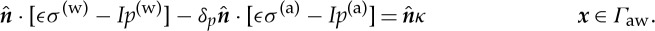
Here, the non-dimensional stress tensors are given by *σ*^(w)^=(**∇*u***^(w)^)+(**∇*u***^(w)^)^T^, *σ*^(a)^=(**∇*u***^(a)^)+(**∇*u***^(a)^)^T^.

We note that the scaling of the air velocity and pressure is different to the scaling on the water velocity and pressure by a factor of *ϵ*. This is justified both physically and mathematically. Physically, it can be seen using equation (2.3g) that it takes an air velocity of order *ϵ*^−1^ to induce a water velocity of order 1. Mathematically, it can be seen that scaling the velocity of air and water identically would lead to the replacement of *δ*_*u*_ by *ϵδ*_*u*_ in equation (2.3g) and *δ*_*p*_ by *ϵδ*_*p*_ in equation (2.3h). The result of this change is that it is impossible to balance the equations at *O*(1) when the homogenization procedure is applied.

The *ϵ* in front of the scaled gravitation term in equation (2.3b) comes from the difference in scaling of the water and air velocities. This choice of scaling is appropriate for the flow of air and water in soil. However, the method described here is widely applicable for Stokes flow and it is trivial to adapt the resulting equations for different gravitational scaling.

### (b) Microscale behaviour

We start by considering how the microscale geometry affects flow on the aggregate scale. In order to do this, we seek an asymptotic solution to the scaled equations (2.3). We do this first in terms of *ϵ* to isolate the flow behaviour in the aggregate scale unit cell (figure 1*b*), then in terms of *η* to isolate the flow behaviour in the microscale unit cell (figure 1*c*). Initially, we define two different spatial scales ***x*** denotes the macroscale spatial coordinate, ***y***=*ϵ****x*** denotes the aggregate scale spatial coordinate. Expanding the velocity and pressure in powers of *ϵ* we obtain
2.4
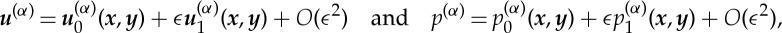
where *α*={*w*,a} denotes the water and air phase, respectively, and apply the two-scale homogenization procedure [3], **∇**=**∇**_*y*_+*ϵ***∇**_*x*_. Collecting terms at order *ϵ*^0^ we obtain
2.5a
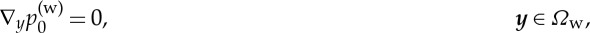

2.5b
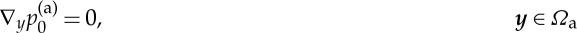

and
2.5c
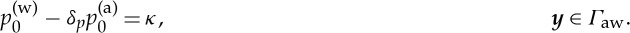


Equations (2.5) have solution, 
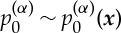
, i.e. the leading order pressure is a function of the macroscopic variable only. Furthermore, equation (2.5c) relates the macroscopic air and water pressures through the surface tension curvature product. In order to determine the effect of the macroscopic pressure gradients on the microscale flow, we proceed to the next order of the expansion and obtain a set of equations for the leading order velocity and the order *ϵ* pressure correction
2.6a
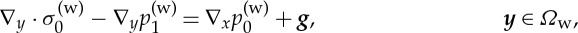

2.6b
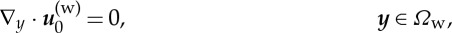

2.6c
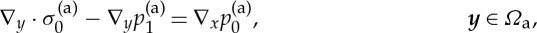

2.6d
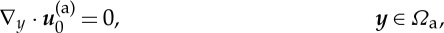

2.6e
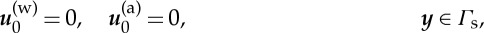

2.6f
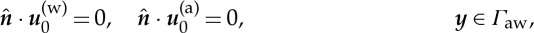

2.6g
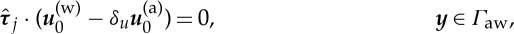

2.6h
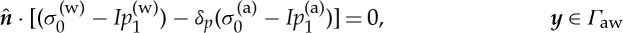

and
2.6i
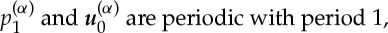
where 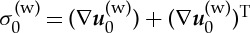 and 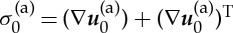. At this point, we could solve equations (2.6) to obtain the macroscopic hydraulic conductivity of the soil. However, as the aggregate is made up of small particles with tiny pores, this would be computationally intensive. To overcome this, we search for approximate solutions to equations (2.6) which average out the flow in the aggregate.

As the structure in the aggregate is periodic with period *η*, we introduce the new variable ***z***=***y***/*η* to denote the microscopic spatial coordinate and expand the gradient operator as **∇**_*y*_=(1/*η*)**∇**_*z*_+**∇**_*y*_. As a result, we have three different spatial coordinates capturing the three different spatial scales ***x***, ***y*** and ***z*** to denote spatial position on the macroscale, the aggregate scale and the microscale, respectively.

For simplicity, we assume that the aggregate is completely saturated with water. Hence, we need only consider one-phase flow in this region. We denote the aggregate domain *Ω*_d_, the extra-aggregate water domain as *Ω*_*ws*_=*Ω*_w_\*Ω*_d_ and expand the Darcy domain velocity and pressure in *η*
2.7a
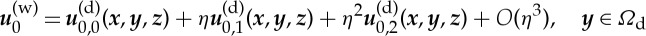
and
2.7b
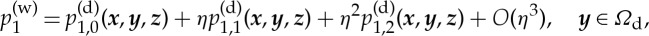
with the fluid equations
2.8a
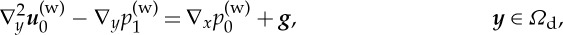

2.8b
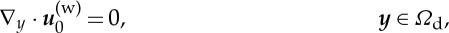

2.8c
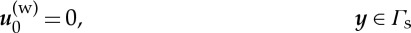

and
2.8d
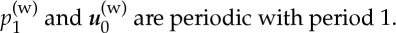
We are interested in writing an averaged equation which describes the behaviour of the fluid on the microscale. The microscale geometry may be considered periodic assuming that we are far from the aggregate boundary. To obtain an approximation for the flow in this domain, we substitute equations (2.7) into (2.8) and consider a single periodic unit cell on the microscale (figure 1*c*). Collecting the terms in equation (2.8) in ascending powers of *η*, we obtain 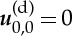, 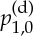 is invariant on the microscale, i.e. 
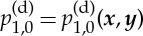
 and ***u***^(d)^_0,1_=0. We note that ***u***^(d)^_0,0_=0 and ***u***^(d)^_0,1_=0 effectively rescales the Darcy velocity to *O*(*η*^2^). However, in order that the velocities internal and external to the aggregate remain scaled the same we retain the original scaling. We expand to order *η*^0^ to obtain the well-known result for single-phase Darcy flow, see for example [4], with an additional source term owing to the ***x*** dependence of the pressure gradients
2.9a
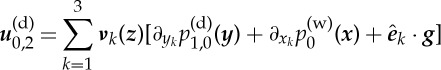
and
2.9b
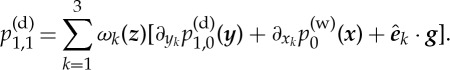
Here, 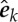 is a unit vector in the *k*th direction and ***ν***_*k*_ and *ω*_*k*_ satisfy the cell problem
2.10a
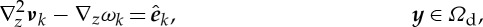

2.10b
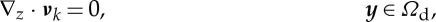

2.10c
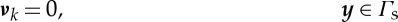

and
2.10d
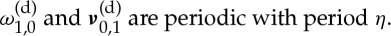
Expanding equation (2.8b) to *O*(*η*), integrating over the Darcy domain, applying the divergence theorem and using equation (2.9), we derive the averaged equation for the microstructure
2.11
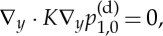
where *K* is the hydraulic conductivity of the aggregate,
2.12
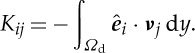
This result allows us to apply Darcy's law on the interior of the aggregate with the microscale flow driven by the macroscopic pressure difference. However, in order to obtain the total macroscopic flow in the medium, we need to consider flow on the aggregate scale. Before we can do this, we need to define appropriate boundary conditions for the flow at the interface between the aggregate, in which Darcy flow holds, and the adjacent pore space, in which Stokes flow holds.

### (c) Boundary condition

In order to complete the approximation for the hydraulic conductivity on the aggregate scale, we consider the boundary condition on the aggregate surface. We follow the method of Jäger & Mikelic [19–22].

The key observation is that the Darcy velocity in the aggregate scales with 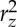, where *r*_*z*_ is the pore radius while in the pore space the velocity scales with 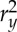, with *r*_*y*_ the radius of the macropores external to the aggregate. Typically, we expect that the ratio of these numbers *r*_*z*_/*r*_*y*_≈*L*_*z*_/*L*_*y*_=*η* such that ***u***^(*d*)^∼*O*(*η*^2^) and ***u***^(w)^∼*O*(1). Using this assumption, we define the rescaled average aggregate velocity
2.13
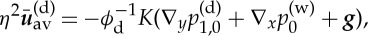
where 
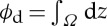
 is the microscale porosity defined as the total fraction of the representative microscale volume available for flow. In the previous section, we derived Darcy's law for flow through the aggregate. This allows us to write the equations on the aggregate scale as
2.14a
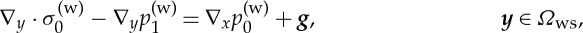

2.14b
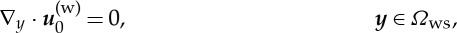

2.14c
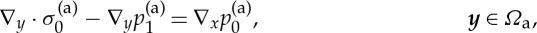

2.14d
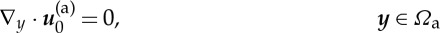

and
2.14e
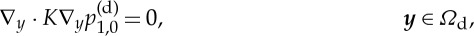
where the boundary conditions (2.6*f*–*h*) still hold. We now determine the behaviour of the flow at the interface between the aggregate and the adjacent pore space. We assume that the aggregate surface is strongly hydrophillic and, as such, the air–water interface remains at a distance from the interface which may be considered large on the microscale. This allows us to derive the boundary conditions assuming that only water is in contact with the aggregate. We define a false interface denoted *S* and, as we did in §2*b*, expand the fluid velocity and pressure outside the aggregate in powers of *η*
2.15a
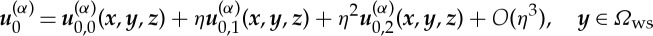
and
2.15b
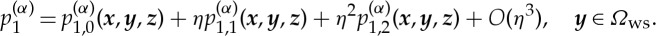
Our aim is now to match the Darcy velocities and pressures to the pore space velocities and pressures of equal order in *η* on *S*. Substituting (2.15) into (2.14) and collecting terms of order *η*^0^, we obtain the Stokes problem for the velocity and pressure of the water and air phases
2.16a
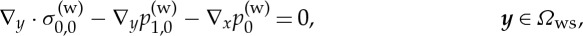

2.16b
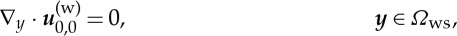

2.16c
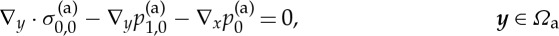

and
2.16d
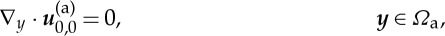
where 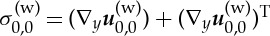 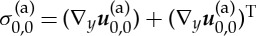

2.16e
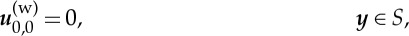

2.16f
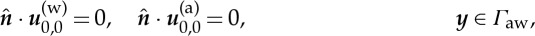

2.16g
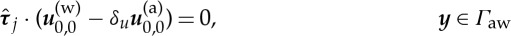

and
2.16h
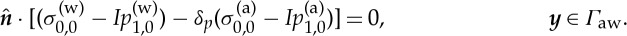
The assumption of no slip at the interface induces a jump discontinuity in the shear stress which is equal to 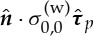, for *p*={1,2}. The presence of a shear stress at such a false interface is clearly unphysical. To correct for this, we consider the behaviour of the water in the region close to the aggregate surface. Specifically, we consider a boundary layer of width ∼*O*(*η*) and, as in §2*b*, rescale ***y***=*η****z***, **∇**_*y*_=*η*^−1^**∇**_*z*_, and look for a velocity and pressure which balance the shear stress jump in this region. Expanding the velocity and pressure in the boundary layer we find that the velocity balances at order *η* and the pressure at order 1:
2.17
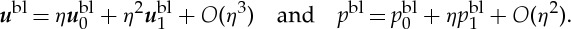
The boundary layer problem which results from expansion (2.17) is
2.18a
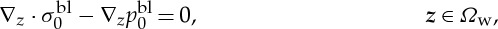

2.18b
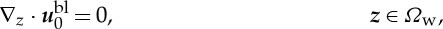

2.18c
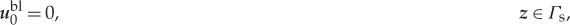

2.18d
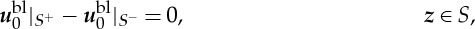

2.18e
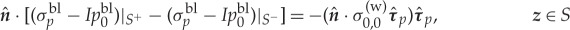

and
2.18f
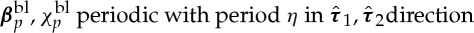
with 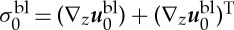. Equations (2.18) are separable; hence, we can write the velocity and pressure local to the interface as
2.19
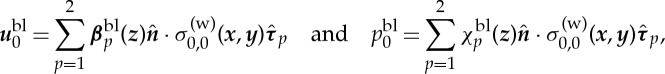
where 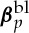 and *χ*^bl^_*p*_ satisfy the cell problem
2.20a
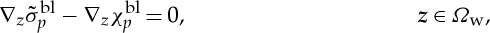

2.20b
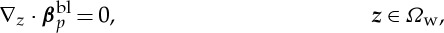

2.20c
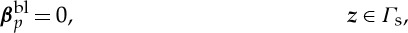

2.20d
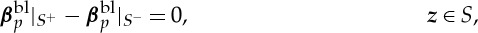

2.20e
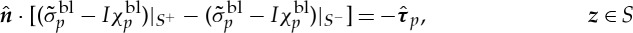

and
2.20f
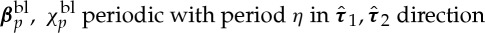
and 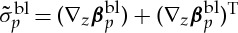. Here, *S*^+^ denotes the water side and *S*^−^ denotes the aggregate side of the false boundary and 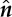 is a normal vector pointing out of the aggregate. As equation (2.20) is defined on an infinite domain in the direction normal to *S*, the solution method is non-trivial. Results from [21] conveys that the velocity tends to zero with distance from the false boundary in *Ω*_d_ and that the normal velocity tends to zero with distance from the boundary in *Ω*_w_. However, the tangential velocity tends to a constant in *Ω*_w_. Furthermore, the pressure has non-zero average at the boundary which will induce an additional flow in the aggregate. We define the far field velocity, 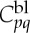, and the pressure jump, 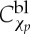, as
2.21
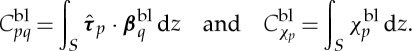
In order to solve these equations on a finite-sized geometry, Jäger *et al*. [21] propose a set of additional constraints which enforce the predicted far field behaviour of the flow. Specifically, as the velocity tends to zero with distance from the false boundary in *Ω*_d_ we apply a no-slip condition at a finite distance, 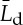, from the false interface. In the Stokes domain, the normal velocity tends to zero with distance from the false boundary and the tangential velocity tends to a constant. Hence, we apply a slip boundary condition at a distance 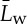 from the false boundary
2.22a
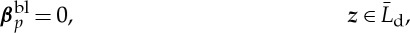

2.22b
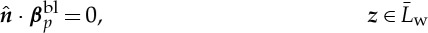

and
2.22c
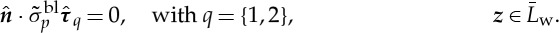
As these conditions enforce the predicted behaviour, we expect that as 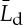 and 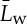 increase the solution will converge. Typically, this happens for 
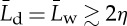
.

As ***β***^bl^_*p*_ decays to a non-zero constant with distance from *S*, the solution extends into the bulk and must add an additional contribution to the flow. This counter flow is generated in the aggregate scale pore space along with an additional pressure term at the boundary which contributes to the flow in the aggregate. Specifically, the additional flow satisfies Stokes equations in the water and air domains
2.23a
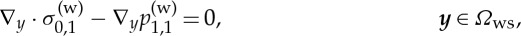

2.23b
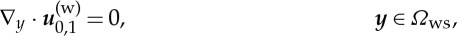

2.23c
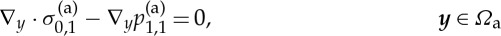

and
2.23d
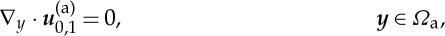
where 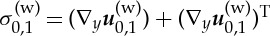 and 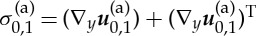. The additional velocity at the false boundary must be matched to the velocity in the pore space
2.23e
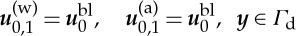
and the first-order correction to the air–water boundary conditions in *η* are
2.23f
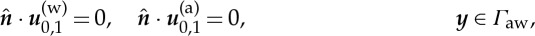

2.23g
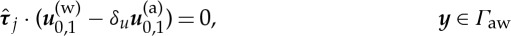

and
2.23h
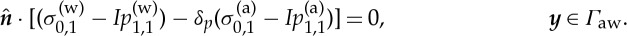
In the aggregate Darcy's law, derived in §2*b*, holds with the boundary pressure given by the pressure in the fluid domain and the additional correction from the boundary layer
2.24a
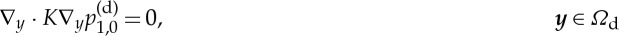
and
2.24b
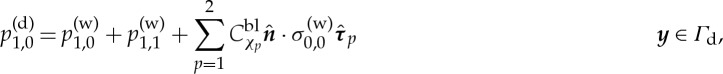
where we recall that the microscale hydraulic conductivity *K* is defined by equation (2.12). The next order correction comes from the Darcy velocity at the boundary. As the Darcy velocity is small, 
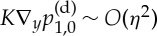
, it will induce a correction ∼*O*(*η*^2^) in the boundary layer. We write the correction to the boundary layer problem as follows:
2.25a
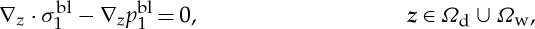

2.25b
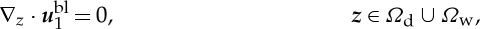

2.25c
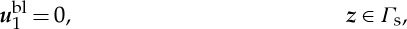

2.25d
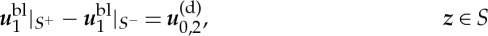

and
2.25e
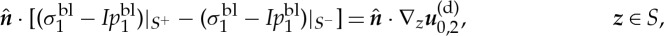
where 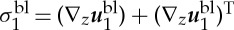. As in the leading order boundary layer problem we could solve equations (2.25) to obtain the velocity near the boundary and consider the counter flow generated in the pore space. However, as the modulated part of the solution decays with distance from the boundary and the constant part is zero in the Darcy region we expect that the velocity in the pore space will stabilize to the average velocity at the interface of the porous medium.

The average velocity at the interface is simply the Darcy velocity divided by the aggregate porosity *ϕ*_d_, see equation (2.13). Therefore, the *O*(*η*^2^) counter flow will be generated directly by the Darcy velocity. We note that the stress contribution on the particle scale is the integrated average of the normal derivative of the tangential velocity on the microscale. This is identically zero and the stress jump from the flow in the Darcy region does not directly contribute to the flow in the pore space. The counter flow generated by the Darcy velocity is given by
2.26a
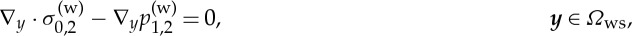

2.26b
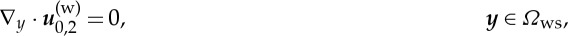

2.26c
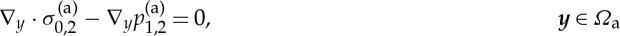

and
2.26d
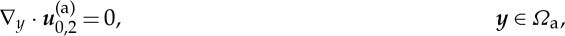
with 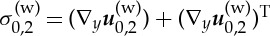, 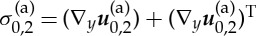 and the boundary conditions
2.26e
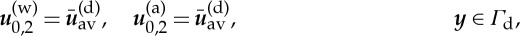

2.26f
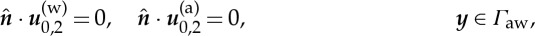

2.26g
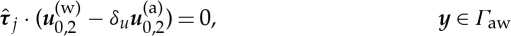

and
2.26h
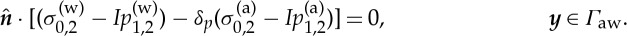
Rather than solve the hierarchy of cell problems which generate the flow in the aggregate and the adjacent pore space, it is computationally more efficient to use the behaviour in the boundary layer to write an averaged boundary condition on the aggregate scale. By combining the contributions at different orders, we can write the boundary conditions on the false boundary as follows:
2.27a
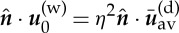

2.27b
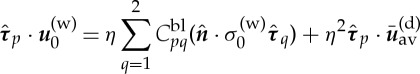

and
2.27c
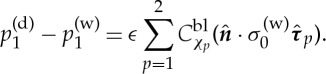
Clearly, these conditions are only correct to *O*(*η*^2^) and more terms must be considered if higher accuracy is needed.

As we are only interested in the leading order solution to the homogenized problem, the maximum order of terms considered in the boundary condition must depend on the ratio *η*/*ϵ*.

If *η*∼*O*(*ϵ*), then the boundary layer terms correspond to a correction order *ϵ* and need not be considered at this order. If this is the case then the appropriate condition on the aggregate boundary is the no-slip condition. For the case where *η*^2^∼*O*(*ϵ*) then the first-order correction in *η* must be considered and the appropriate boundary condition is the Saffman condition. Finally, if *η*^3^∼*O*(*ϵ*), then the appropriate boundary condition is the original Beavers and Joseph condition. We will revisit this point in §4.

### (d) Aggregate scale

We now return to the equations for the macroscale hydraulic conductivity, i.e. the flow on the ***x*** scale (figure 1a). Using the aggregate scale hydraulic conductivity from §2*b* and the boundary conditions from §2*c*, we can write equations (2.6), for all orders in *η*, as
2.28a
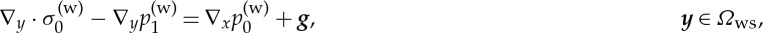

2.28b
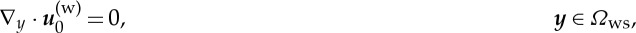

2.28c
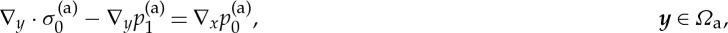

2.28d
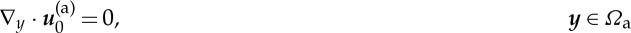

and
2.28e
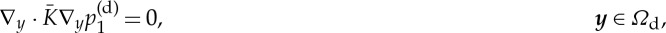
with the boundary conditions at the air–water interface
2.28f
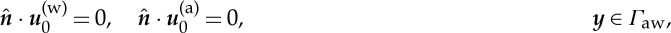

2.28g
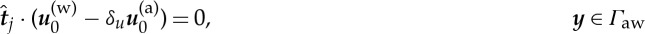

and
2.28h
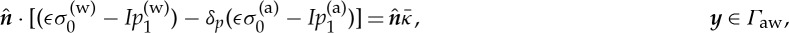
and the Stokes–Darcy interface
2.28i
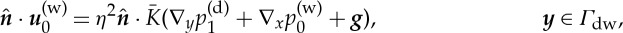

2.28j
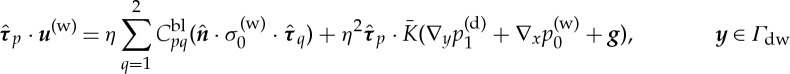

and
2.28k
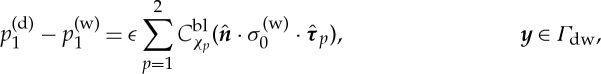
where 
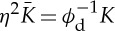
 and 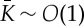. Equations (2.28) are separable and, hence, the solutions can be written as
2.29
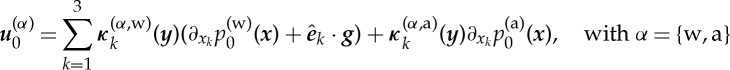
and
2.30
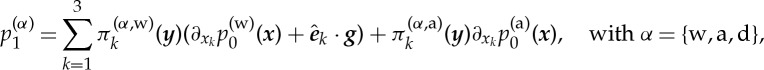
where 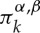 and 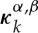 satisfy the cell problems for *β*={a,*w*}
2.31a
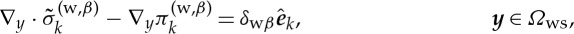

2.31b
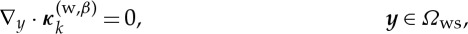

2.31c
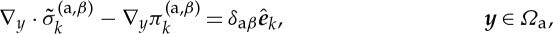

2.31d
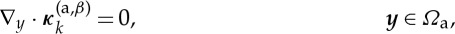

2.31e
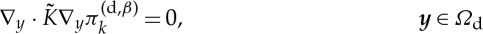

and
2.31f
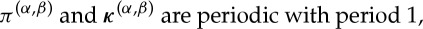
with 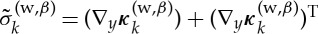, 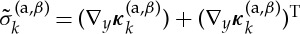 and the air–water boundary conditions
2.31g
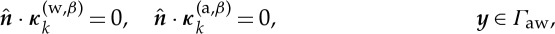

2.31h
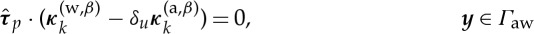

and
2.31i
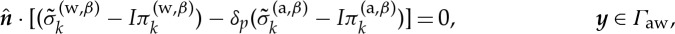
and aggregate boundary conditions
2.31j
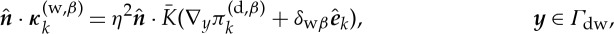

2.31k
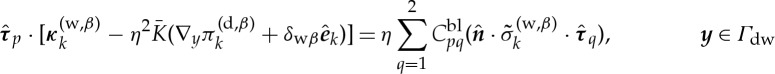

2.31l
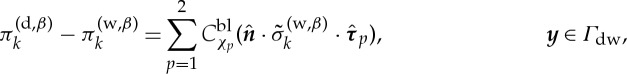
where *δ*_*αβ*_=1 for *α*=*β* and *δ*_*αβ*_=0 otherwise. The additional source terms in equations (2.31*j*,*k*) come from the homogenization procedure on the aggregate surface. Physically, these terms provide the hydraulic conductivity contribution owing to the macroscopic pressure gradient across the aggregate. We also obtain through the first-order correction and application of the Fredholm alternative the macroscopic Richard's equation for saturated flow
2.32a
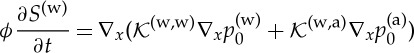
and
2.32b
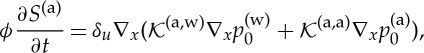
where 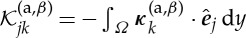 and 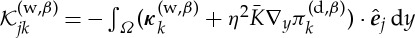.

Equations (2.32), the capillary pressure equation (2.5c) and the saturation condition *S*^(w)^+*S*^(a)^=1 combined with the cell problems described in §2*b*,*c* describe the pressure-driven saturation of soil in a vuggy porous medium with the hydraulic conductivity parameters determined entirely based on the aggregate geometry.

This model captures the flow of two fluids in and around the aggregate for the case in which the aggregate is strongly hydrophilic. This model can be simplified in the case of single-phase flow. In this case, *S*^(w)^=1 and we can see that equations (2.31) simplify to give a single cell problem for ***κ***^(*w*,*w*)^ and *ω*^(*w*,*w*)^
2.33a
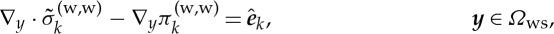

2.33b
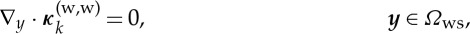

2.33c
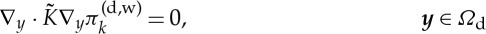

and
2.33d
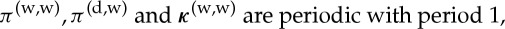
with the aggregate boundary conditions
2.33e
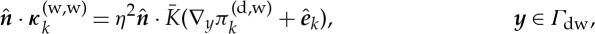

2.33f
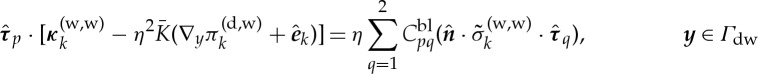

and
2.33g
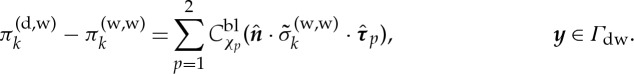
The result of this simplification is that 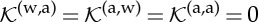 and, neglecting terms *O*(*η*^2^), equation (2.32) reduces to the standard Darcy's law for vuggy porous media [10]. However, we should note that the constant in the Saffman approximation is derived from the geometry rather than left as a fitting parameter. Similarly, in the limit that the porosity of the aggregate goes to zero, we can recover the two-phase flow models described in [4] simply by neglecting terms *O*(*η*) in equations (2.31).

The theory derived in this section highlights the role of the different space scales in the derivation of the macroscale hydraulic conductivity in soils. Physically, the relative sizes of *η* and *ϵ* conveys the macroscopic length scale at which the assumptions made on the aggregate boundary are applicable within the approximations made in the aggregate scale homogenization procedure. The macroscopic length scale can be written 
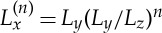
, where 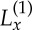 is the length scale on which the no-slip condition may be used on the aggregate surface and the higher order terms do not contribute significantly to the flow. The length scale 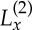 tells us the length at which Saffman's simplification of the Beavers and Joseph boundary condition produces a contribution to the flow of order 1, 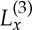 is the length scale at which the fully coupled model must be solved.

## 3. Example

In this section, we numerically study the effect of the boundary condition used on the macroscopic hydraulic conductivity. We consider two cases both of which are axially symmetric about the ‘false’ boundary normal. In this case it can be proved that the pressure correction 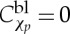 [21]. In §3a, we study the hydraulic conductivity of a two-dimensional system with different aggregate structures. In §3*b*, we consider the effect of the aggregate structure on a three-dimensional idealized soil sample.

### (a) Two-dimensional geometry

**Figure 2.**
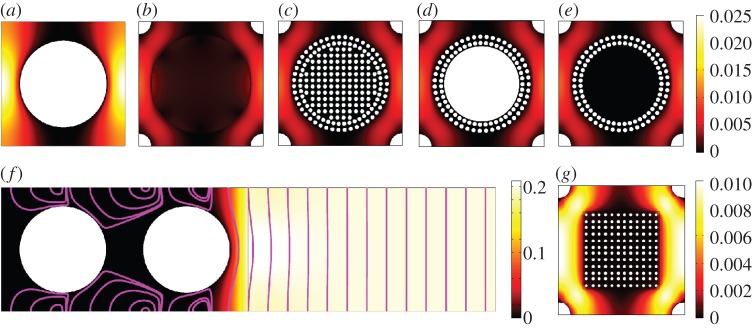
Numerical solutions showing absolute dimensionless velocity. (a) Shows the microscale cell problem, (*b*) the numerical solution with the geometrically derived Beavers and Joseph boundary condition, (*c*) the full numerical solution with the aggregate structure taken into account, (*d*) shows the solution for an aggregate with a solid core and (*e*) shows the solution for an aggregate with a hollow core, (*f*) shows the solution in the boundary layer with velocity streamlines and (*g*) shows the full numerical solution for the square aggregate geometry. (Online version in colour.)

We consider two different two-dimensional geometries using the method illustrated in figure 1. The first geometry consists of a periodic packing of circular aggregates with radius 0.35*L*_*y*_. The second geometry we have considered for the two-dimensional case is a square aggregate (figure 2*g*). The square is aligned to the principal coordinate axis, has side length 0.4*L*_*y*_ and has smoothed corners with radius of curvature 0.05*L*_*y*_. The microscopic aggregate structure is also periodic and is composed of particles which are ellipses with principle radii *r*_*τ*_=*R*_*τ*_*L*_*y*_*η* and *r*_*n*_=*R*_*n*_*L*_*y*_*η* and {*R*_*n*_,*R*_*τ*_}∈[0,0.5] with *R*_*τ*_=0.5 or *R*_*n*_=0.5 corresponding to the particles touching. Here, the particle scale is *η*=0.05, the resulting minimum micropore radius is given by min{(1−2*R*_*n*_)*ηL*_*y*_/2,(1−2*R*_*τ*_)*ηL*_*y*_/2} .

The cell problem for aggregate hydraulic conductivity, (2.31), depends only on the parameters *δ*_*p*_ and *δ*_*u*_. These are determined by the viscosity and density of the two fluids and the ratio of the macroscopic-to-microscopic length scales. In our simulations, we have used *μ*^(w)^=10^−3^ Pa s, *μ*^(a)^=1.8×10^−5^ Pa s, *ρ*^(w)^=10^3^ and *ρ*^(a)^=1.2. The typical aggregate size is *L*_*y*_=10^−3^ m [37], and we consider a macroscopic length scale of *L*_*x*_=10^−1^ m, chosen to be comparable to length scales for soil columns used in X-ray CT imaging [36]. The resulting dimensionless parameters are *δ*_*p*_=8.47 and *δ*_*u*_=0.15.

There is clearly a question as to the applicability of the boundary condition on the curved surface of the aggregate and, for the circular aggregate, the periodicity of the particles inside. Here, we assume that the boundary conditions derived in §2*c* are appropriate and that the errors induced by this assumption will be small.

We calculate the boundary conditions and the macroscopic hydraulic conductivity for a variety of different-sized and -shaped particles within the aggregate using Comsol Multiphysics. The Saffman problem decouples and the Stokes problem is solved in isolation before the Darcy velocity is calculated in the aggregate. For the Beavers and Joseph case, the equations are solved iteratively in *η*. First, the Stokes problem is solved, then the output is used as a boundary condition for the Darcy problem, this is then used to calculate the correction to the Stokes problem. We do this for four cases: the case where the full geometry is taken into account, the case where a no-slip condition is applied to the surface of the aggregate, the case where only the Saffman condition is applied and the case where the full Beavers and Joseph condition is applied (figure 2). In addition, to verify the role played by the internal aggregate structure, we have calculated the hydraulic conductivity without approximation for both aggregate shapes with a solid core and with a hollow core (see figure 2*d*,*e* for the geometry in the circular case). The full geometry case involves solving equations (2.6), the various approximations involve solving equations (2.31) neglecting terms of order *η* for the no-slip case, *η*^2^ for the Saffman case and order *η*^3^ for the Beavers and Joseph case.

The boundary slip tensor *C*^bl^_*pq*_ has size 1 is given for a variety of different ellipsoidal particles in table 1. It is clear from these results that the radius in the direction normal to the boundary has significantly more effect than the radius tangential to the boundary. Physically, this is expected as the boundary layer problem is calculating the response of the tangential velocity to the surface geometry. Increasing the particle radius in the normal direction reduces the size of the flow pathways within the aggregate resulting in a large decrease in the flow. Conversely, increasing the particle radius in the tangential direction leaves the critical flow pathway unchanged and has relatively little effect on the flow.

**Table 1.** Calculated values for 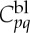 in the two-dimensional case for elliptic particles with principle radii *R*_*τ*_ and *R*_*n*_.

*R*_*τ*_\*R*_*n*_	0.10	0.15	0.20	0.25	0.30	0.35	0.40	0.45
0.10	0.472	0.423	0.374	0.325	0.276	0.227	0.177	0.128
0.15	0.461	0.414	0.366	0.318	0.269	0.220	0.171	0.122
0.20	0.451	0.405	0.358	0.310	0.262	0.214	0.165	0.117
0.25	0.442	0.397	0.351	0.304	0.256	0.208	0.160	0.111
0.30	0.434	0.390	0.344	0.298	0.250	0.203	0.155	0.106
0.35	0.428	0.384	0.339	0.292	0.245	0.198	0.150	0.102
0.40	0.423	0.379	0.333	0.287	0.240	0.193	0.145	0.097
0.45	0.419	0.375	0.329	0.283	0.236	0.189	0.140	0.093

**Figure 3.**
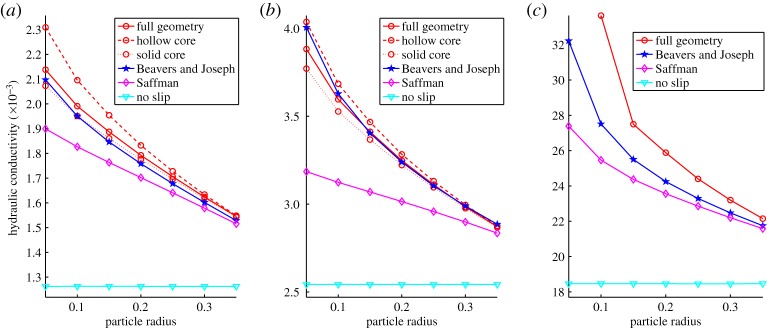
Numerically calculated macroscale dimensionless hydraulic conductivity for the full model, the case with the Saffman approximation and the case with the full Beavers and Joseph approximation in (a) two-dimensional circular, (*b*) two-dimensional square and (*c*) three-dimensional circular cases. (Online version in colour.)

The results for the macroscale dimensionless hydraulic conductivity are plotted for the square and circular aggregates in figure 3 for the circular inter-aggregate particles. These results show that the successive approximations for the boundary conditions produce higher accuracy results. As the microparticle radius is increased and, hence, the pore size is decreased the results converge towards the no-slip value. In the limit that the micropore size tends to zero, there is zero connectivity through the aggregate and the Darcy velocity must vanish, note this is not the case in three dimensions. The result is that the Saffman and the Beavers and Joseph conditions will converge to the same solution.

Comparison between the aggregates with different internal geometries (figure 2*c*–*e*) shows that the internal structure of the aggregate has little effect on the hydraulic conductivity for larger particle sizes (figure 3) this trend is observed for both the square and circular aggregates. This is to be expected based on the accuracy of the Beavers and Joseph and the Saffman approximations at larger particle radius where the internal structure of the aggregate only comes into play at *O*(*η*^2^).

The no-slip approximation which neglects all internal structure of the aggregate provides a poor approximation of the fluid properties. This improves with decreasing pore radius; however, it still performs poorly in comparison to the Beavers and Joseph or the Saffman conditions. In the case of the circular and spherical aggregates, the Saffman approximation, which includes the effects of the *O*(*η*) boundary layer, is significantly better than the no slip and reproduces the trends in the hydraulic behaviour as the microparticle size changes. It also provides a good quantitative approximation for the hydraulic conductivity. The Saffman approximation provides a less accurate approximation in the case of the square aggregate which is particularly notable for particle radius less than 0.25. This is because of the sharp corners present in the square geometry which induces a high-pressure gradient in the Stokes region. This results in a large Darcy velocity on the aggregate corner which decays rapidly with distance into the aggregate but induces a sizable counter flow in the Stokes domain at the corner of the aggregate. This counter flow is neglected in the Saffman approximation, as it comes into play at *O*(*η*^2^). However, it is clearly needed to capture the effects of the corner at high aggregate porosity.

The fully coupled case in which the flow in the aggregate is computed using Darcy's law coupled to the flow in the external pore space provides the best approximation. The boundary conditions and behaviour inside the aggregate are derived assuming periodicity of the structures. While the microscale inside the aggregate is clearly periodic the assumption of periodicity on the boundary is less accurate. The approximations used in the derivation require a flat interface with a periodic structure of microparticles. The interface curvature and the conflicting requirements for both a periodic structure inside the aggregate and on the aggregate surface leads to errors in the approximation. However, these are small even in the worst case, ≈2% for the Beavers and Joseph condition.

There is a clear advantage of using approximate boundary conditions rather than studying the full geometry in terms of computational speed. The full Beavers and Joseph approximation to the boundary condition is also clearly more accurate than the Saffman condition for small particle radius. However, the consequence is that the fully coupled model has to be solved for flow around and within the aggregate.

### (b) Three-dimensional geometry

The advantage of approximation techniques is clearly greater in three-dimensional geometries where computation times can be large. Here, we consider the three-dimensional extension of the aggregate considered in §3a. The aggregate is spherical with radius 0.35*L*_*y*_, the microparticles are spherical particles of radius *RL*_*y*_*η* and again *η* is taken as 0.05. The internal structure of the aggregate is formed from spherical particles packed to form a cubic lattice with a shell, two layers thick, of spheres around the outside. Packing the particles evenly within the shell proves to be a much harder problem as there is no possible particle arrangement which provides an even distribution of spheres. However, in order that these methods provide a reasonable description of fluid flow in non-ideal geometries, it is essential that they are insensitive to small changes in the periodicity at the surface. Therefore, we have made no attempt to minimize the inhomogeneity in the sphere distribution and show numerically that the resulting hydraulic conductivity is still accurate.

The macroscopic hydraulic conductivity is calculated for a range of different microparticle sizes, for each of the different cases considered in the two-dimensional example, using Comsol Multiphysics for the approximate cases and OpenFoam for the case in which the full geometry is taken into account. The equations implemented in OpenFoam are solved using the SIMPLE (Semi Implicit Method for Pressure-Linked Equations) algorithm [38], with an added source term. As the particles are spherical the diagonal components of the stabilization tensor are equal, 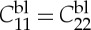 the off diagonal components are zero 
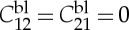
. The value of *C*^bl^_11_ is given for different *R* in table 2.

**Table 2.** Calculated values for 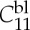 in three-dimensional case for spherical soil particles of radius *R*.

*R*	0.05	0.10	0.15	0.20	0.25	0.30	0.35	0.40	0.45
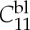	1.033	0.730	0.589	0.493	0.415	0.347	0.282	0.220	0.161

**Figure 4.**
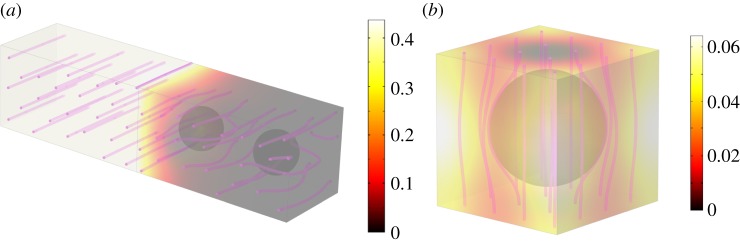
(a) Typical solutions to three-dimensional boundary layer problem and (*b*) aggregate scale cell problem with the Beavers and Joseph boundary condition showing absolute dimensionless velocity and velocity streamlines. (Online version in colour.)

We now proceed as in the two-dimensional case and calculate the hydraulic conductivity for the four different cases. Typical solutions to the cell problems are shown in figure 4 and the results of these calculations are shown in figure 3. Both the Beavers and Joseph and the Saffman conditions offer a significant improvement in the estimated hydraulic conductivity when compared with the no-slip condition and highlight the importance of the approximate modelling techniques used in this paper. In terms of computational time, the approximate solutions took approximately 5 min to calculate for each radius on a desktop PC. The calculation which takes the full geometry of the aggregate took 20 h for each radius value running on 32 nodes of the IRIDIS high-performance computing facility at the University of Southampton.

For low particle radii, corresponding to large pore sizes, the three-dimensional approximation behaves poorly with ≈20% error for *R*=0.1. This is not unexpected as, owing to the large pore sizes, there is significant flow through the aggregate and the errors induced by the disordered sphere packing and the surface curvature will become significant. However, it can be seen that for large particle radii, corresponding to small pore size, the error is significantly reduced to ≈9% at *R*=0.15 and ≈2% for *R*=0.35.

The accuracy of the approximation techniques for large radius values, corresponding to small pore sizes, tells us that the internal aggregate structures is largely irrelevant in determining the macroscopic hydraulic conductivity. Rather, it is the pore structure on the surface of the aggregate that makes the most difference to the macroscopic properties of the structure. Hence, it is the surface roughness rather than the microstructure inside the aggregate which should be the focus of imaging techniques in order to obtain accurate solutions from image-based models.

## 4. Summary

In this paper, we have used the method of Jäger & Mikelic [19–22] to derive the Beavers and Joseph boundary condition applicable to the surface of a soil aggregate in a periodic geometry. The resulting equations show how the hydraulic conductivity properties on a macroscopic scale relate to the geometry on the particle scale and the aggregate scale.

The results show that the surface roughness of the aggregate is the key property of the microscale geometry which determines the hydraulic conductivity of the macroscopic geometry. Hence, this is the region in which the resolution of X-ray CT for image-based modelling has the greatest impact. More accurate approximations which take into account the coupling between the flow in the aggregate and the flow in the extra-aggregate pore space produce a slight improvement in the results at the expense of an increase in computation time, typically the Beavers and Joseph simulations take twice as long as the Saffman approximation using the iterative scheme mentioned in §3.

By considering the different scales of the microparticle geometry, the aggregate scale geometry and the macroscopic scale of interest we have determined criteria for selecting which conditions are most applicable. We find that on small scales the error induced by a no-slip boundary condition on the aggregate surface is negligible. However, on larger scales these errors add up and the more accurate Beavers and Joseph condition or the Saffman approximation must be used.

The applicability of equations (2.20) to curved aggregate surfaces is assumed. However, it is seen that for large micropore sizes this induces notable errors in the approximation. There is clearly scope for improving this approximation based on the curvature of the aggregate surface. The assumption of periodicity is applied to the internal structure of the aggregate, the aggregate surface and the aggregate scale packing. For real geometries obtained from X-ray CT, this assumption is not completely valid. Physically, the error induced by this assumption can be achieved by choosing a sufficiently large aggregate scale geometry which may include multiple aggregates. Validation of this assumption could be achieved by comparing several different regions of a single soil sample obtained from X-ray CT. This would not only validate the theory developed in this paper but also determine the required dimensions *L*_*y*_ and *L*_*z*_ required for accurate estimates of hydraulic conductivity to be obtained.

The modelling in this paper was developed in the context of fluid flow in soil. However, it is applicable to a much wider set of situations in the study of porous media, for example petroleum reservoirs. This work highlights the importance of the role of the different scales in determining the macroscopic properties of fluid flow in porous media and how different aspects of the geometry contribute to these values.
